# Hyaluronic Acid Suppresses the Expression of Metalloproteinases in Osteoarthritic Cartilage Stimulated Simultaneously by Interleukin 1β and Mechanical Load

**DOI:** 10.1371/journal.pone.0150020

**Published:** 2016-03-02

**Authors:** Florian Pohlig, Florian Guell, Ulrich Lenze, Florian W. Lenze, Heinrich M. L. Mühlhofer, Johannes Schauwecker, Andreas Toepfer, Philipp Mayer-Kuckuk, Rüdiger von Eisenhart-Rothe, Rainer Burgkart, Gian M. Salzmann

**Affiliations:** 1 Department of Orthopedic Surgery, Klinikum rechts der Isar, Technical University Munich, Ismaninger Str, 22, 81675 Munich, Germany; 2 Department of Traumatology, Universitätsspital Basel, Spitalstr. 21, 4031 Basel, Switzerland; 3 Division of Lower Extremity Surgery, Schulthess Klinik, Lengghalde 2, 8008 Zurich, Switzerland; Université de Lyon—Université Jean Monnet, FRANCE

## Abstract

**Purpose:**

In patients with osteoarthritis (OA), intraarticular injection of hyaluronic acid (HA) frequently results in reduced pain and improved function for prolonged periods of time, i.e. more than 6 months. However, the mechanisms underlying these effects are not fully understood. Our underlying hypothesis is that HA modifies the enzymatic breakdown of joint tissues.

**Methods:**

To test this hypothesis, we examined osteochondral cylinders from 12 OA patients. In a bioreactor, these samples were stimulated by interleukin 1β (Il1ß) (2 ng/ml) plus mechanical load (2.0 Mpa at 0.5 Hz horizontal and 0.1 Hz vertical rotation), thus the experimental setup recapitulated both catabolic and anabolic clues of the OA joint.

**Results:**

Upon addition of HA at either 1 or 3 mg/ml, we observed a significant suppression of expression of metalloproteinase (MMP)-13. A more detailed analysis based on the Kellgren and Lawrence (K&L) OA grade, showed a much greater degree of suppression of MMP-13 expression in grade IV as compared to grade II OA. In contrast to the observed MMP-13 suppression, treatment with HA resulted in a suppression of MMP-1 expression only at 1 mg/ml HA, while MMP-2 expression was not significantly affected by either HA concentration.

**Conclusion:**

Together, these data suggest that under concurrent catabolic and anabolic stimulation, HA exhibits a pronounced suppressive effect on MMP-13. In the long-run these findings may benefit the development of treatment strategies aimed at blocking tissue degradation in OA patients.

## Introduction

Osteoarthritis (OA) of large joints is a disabling condition with significant and rising incidences due to the demographic development in industrial countries [1]. For symptomatic OA, treatment with non-steroidal anti-inflammatory drugs (NSAIDs) remains the gold standard and is intended to delay a surgical intervention especially in younger patients [2]. However, data from patients with rheumatic diseases shows that treatment with NSAIDs can be associated with potentially severe side effects such as peptic ulcer, renal insufficiency or bleeding [3], hence alternate treatment modalities are wanted for OA patients.

Intraarticular injection of hyaluronic acid (HA), a relatively large glucosaminoglycan and important component of the extracellular matrix (ECM) of hyaline cartilage, represents an alternative treatment for OA [4]. Importantly, HA treatment has few if any side effects, while several randomized controlled clinical trials could confirm a positive effect regarding pain and function [5–7]. It is believed that HA treatment provides instant viscosupplementation to the joint. However, the beneficial effects of HA treatment have been observed for up to 8 months despite a half-life of a few days [8, 9]. These results may indicate disease modifying properties of HA besides sole viscosupplementation [10, 11].

A number of studies have set out to unravel mechanisms of disease modification of HA in OA. Early work by Ishida et al. showed that chondrocytes utilize the HA receptor CD44 to adhere to HA in the extracellular matrix and that this interaction induced signaling events and proliferation [12]. Later, Brun et al. reported that administration of HA resulted in an increased survival of chondrocytes exposed to free-radicals, an effect that was mediated by HA-CD44 signaling [13]. More recent work has fostered the view that HA counteracts several different apoptosis pathways and thus facilitates a chondroprotective effect [14, 15]. That administration of HA directly alters intracellular signaling was shown by Andhare et al. They demonstrated that loss of HA disrupts BMP-7-induced but not TGFβ-induced Smad signaling, and that administration of HA restored the disrupted signaling pathway [16]. With respect to IL1β, which is one of the principle cytokines triggering joint destruction in OA [17], Maneiro et al. reported that HA reduced the IL1β-induced production of nitric oxide (NO) and prostaglandin E2 while not exerting an effect on the basal production of these molecules [18]. The protective effect of HA against the catabolic effect of IL1β is not limited to NO and prostaglandins. Experiments by Ohno-Nakahara et al. and Julovi et al. on cultured articular chondrocytes indicated that HA reduced induction of metalloproteinases 1, 3, and 13 [19–21]. It remains uncertain, however, if HA prevents metalloproteinase expression in OA joints, because, as long as the patient is mobile, these joints are mechanically loaded and hence experience a strong anabolic signal from the load [22, 23]. Therefore, we hypothesized for this study, that under combined IL1β and mechanical stimulation, HA exhibits a suppressive effect on the expression of metalloproteinases. We report here that under these conditions, HA exhibits a pronounced suppressive effect particularly on MMP-13.

## Materials and Methods

### Materials

All reagents were from Sigma Aldrich (Sigma-Aldrich, St. Louis, MO, USA) if not stated otherwise. Hyaluronic acid (Ostenil, 1.2–1.4 x10^6^ Da) was from TRB Chemedica (TRB Chemedica, Haar, Germany). Recombinant human IL1ß was also obtained from Sigma Aldrich.

### Patient Cohort

12 patients with osteoarthritis, 6 with grade 2 and 6 with grade 4 osteoarthritis according to Kellgren and Lawrence (K&L), undergoing total knee arthroplasty were included in this study. This study was approved by the local ethics committee (Ethikkommission der Fakultät für Medizin der Technischen Universität München) and written consent was obtained from each subject prior to inclusion.

### Preparation and Culture of Osteochondral Cylinders from OA Patients

During routine arthroplasty distal femoral bone cuts were harvested from the lateral condyle in knee joints with varus deformity and from the medial condyle in valgus knee joints due to the less affected cartilage.

Explantation was followed by immediate sterile and standardized preparation of 4 osteochondral cylinders with a diameter of 6 mm with an OATS harvesting device (Arthrex, Naples, USA) according to a previously defined pattern. Cylinders were then transferred to 6 well plates and cultivated in low glucose DMEM (Dulbecco’s Modified Eagle Medium) supplemented with 1% vitamins, 1% glutamine, 0.2% dexamethasone, 1% ascorbic 2-phosphate, 1% sodium pyruvate, 1% proline, 1% ITS+1 Liquid Media Supplement at 37°C and 5% C0_2_. Prior to combined catabolic and anabolic stimulation in the bioreactor, samples were cultivated for 4 days to adapt to culture conditions.

### Stimulation with IL1ß and mechanical load

**Fig 1:**
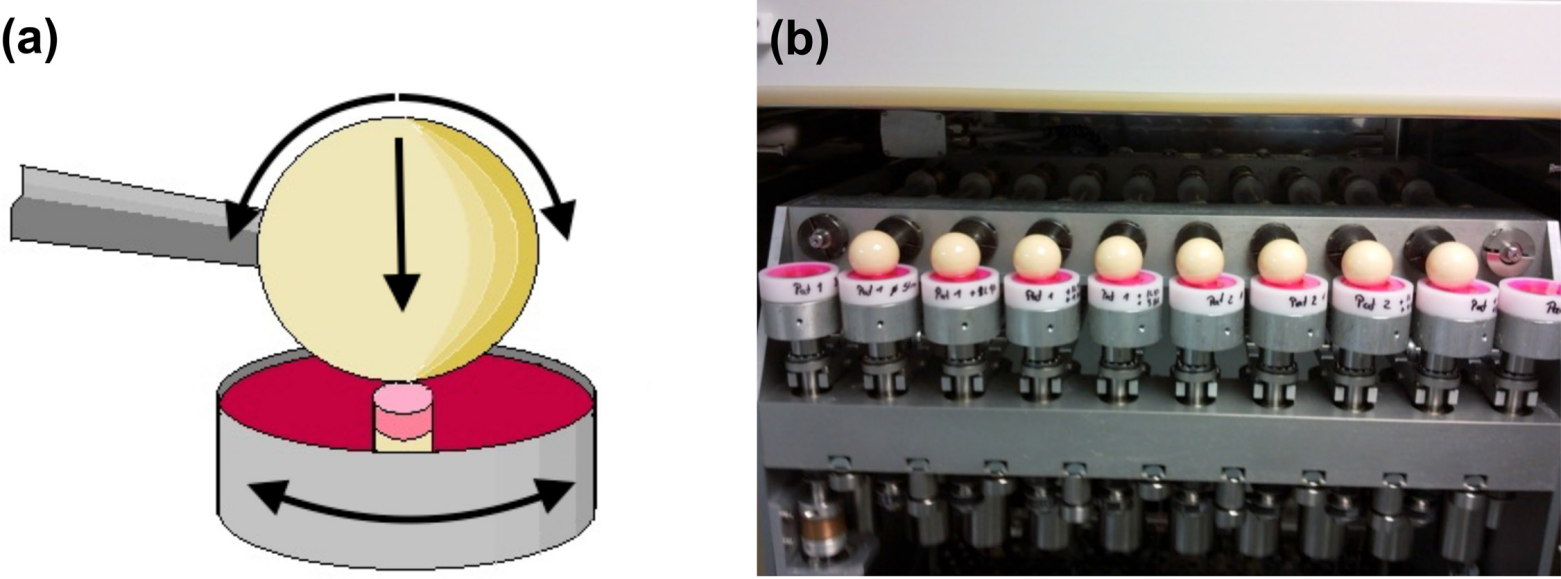
**(a)** Schematic illustration of a sample placed in the chamber and the multi-axial mechanical loading applied in the bioreactor; **(b)** samples in the bioreactor during mechanical stimulation.

After preculturing, the explants were divided into 3 experimental groups: (1) simulation with IL1ß and mechanical load, (2) simulation with IL1ß and mechanical load plus low dose HA, (3) simulation with IL1ß and mechanical load plus high dose HA. The control group received neither mechanical stimulation nor HA. A concentration of 2 ng/ml of IL1ß was used. This concentration has catabolic effects and was previously established [19]. For mechanical loading, a load protocol based on previously published data of 2.0 Mpa was applied at 0.5 Hz horizontal and 0.1 Hz vertical rotation for 2 hours daily and a total of 5 days was chosen [22–25]. Low and high doses of HA were 1 mg/ml and 3 mg/ml, respectively. The control group and each of the 3 experimental groups constituted 12 samples. The bioreactor providing standard cell culture conditions (5% CO_2_, 21% O_2_, 95% humidity and 37°C) and in addition an integrated pin-on-ball system for mechanical loading was utilized for all experiments [26]. For the entire stimulation period the cylinders were fixed in the center of a special sample chamber holding the culture medium. Care was taken to place the subchondral bone entirely into the sample holder leaving only the cartilage exposed to the medium. A total rotation of 15° around its own axis was applied to the sample chamber. Axial force was applied by a ceramic ball with a vertical rotational range of 30° (Fig 1A and 1B). Upon completion of an experiment, samples were shock frosted in liquid nitrogen and stored at -80°C.

### Gene Expression Analysis

Isolation of RNA from the samples was performed with “RNeasy Tissue Kit” (Qiagen, Hilden, Germany) according to the manufacturer`s instructions. In brief, harvested cartilage samples were shredded in a mortar and lysed. RNA from the lysate was isolated on a silica matrix followed by elution with distilled water. Quantification of the harvested RNA was performed photometrically.

Due to the limited amount of harvested RNA a preamplification was performed by reverse transcription using a “High Capacity Reverse Transcription Kit” (Applied Biosystems, Grand Island, USA) according to the manufacturer’s instructions.

In a further step relative quantification of gene expression of MMP-1, -2, -13 was performed by real time-PCR in a thermo cycler with a TaqMan Array and specific primers (MMP-1: Hs00899658_m1; MMP-2: Hs01548727_m1; MMP-13: Hs00233992_m1; GAPDH: NM_002046.3) (Applied Biosystems, Grand Island, USA). Glycerinphosphate-Dehydrogenase (GAPDH) was used as internal control. Each of in total 40 cycles consisted of a 15 second denaturation period at 95°C followed by a combined annealing and extension phase at 60°C for 1 minute.

### Histology

Serial frozen 9 microm sections from the cartilage samples were prepared using a cryotome (Cryostat HM 560, Thermo Fisher Scientific, Waltham, Massachusetts, USA) and mounted. Subsequently, staining with hematoxylin and eosin (H&E) was performed. Adjacent sections were also stained with Fast Green and Safranin O as previously described [27, 28].

### Statistics

Comparative analysis was performed with quantitative PCR data using SPSS software (IBM, Armonk, USA) and the Mann-Whitney-Wilcoxon signed ranked test. The results are shown as mean ± standard deviation. A p-value of ≤ 0.05 was considered statistically significant.

## Results

**Fig 2:**
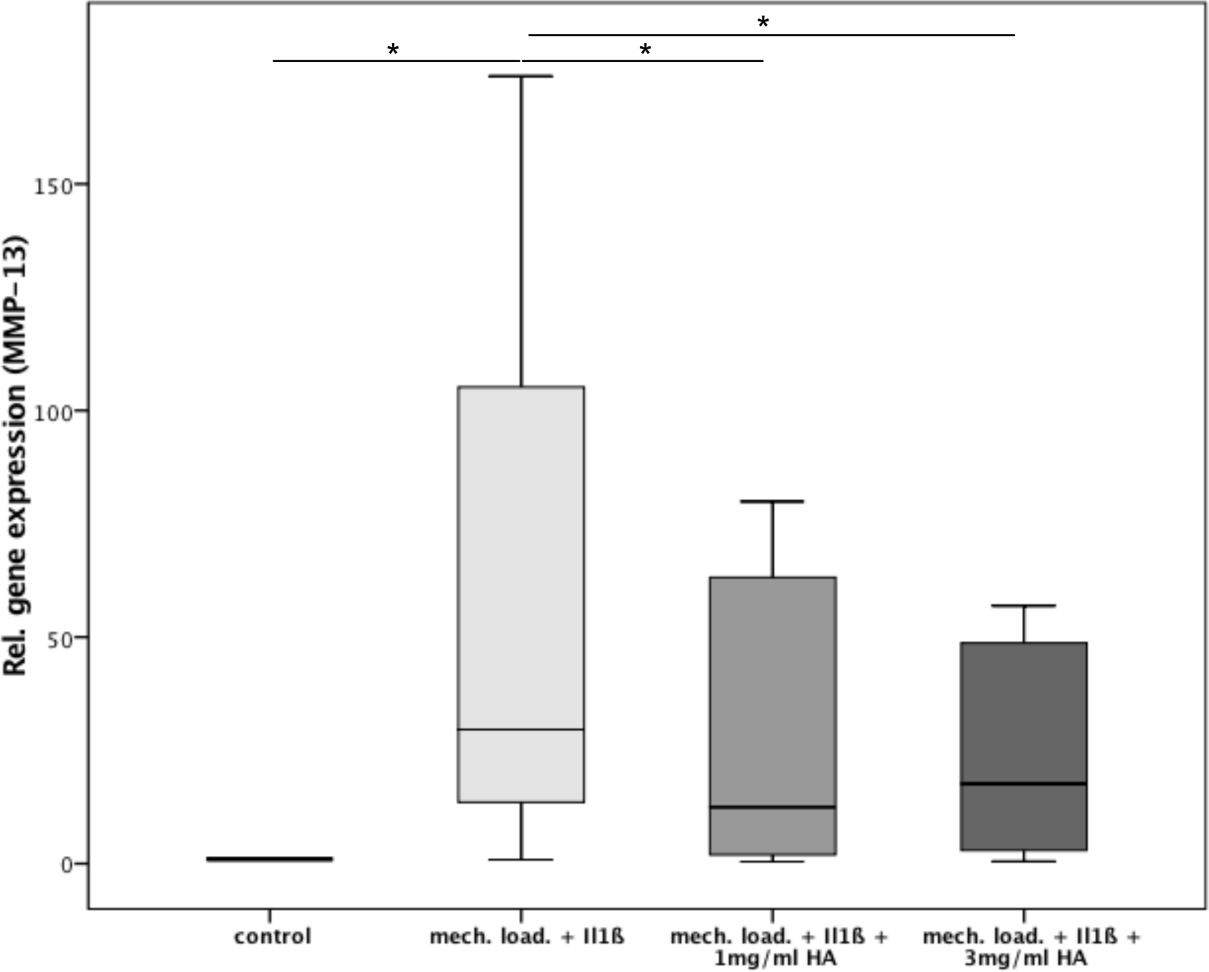
Relative gene expression of MMP-13 in the 3 study groups: (1) 2ng/ml IL1ß + mechanical loading, (2) 2ng/ml IL1ß + mechanical loading + 1mg/ml HA, (3) 2ng/ml IL1ß + mechanical loading + 3mg/ml HA and the control; * indicates statistical significance with p<0,05.

Osteochondral cylinders from patients with OA were stimulated with IL1ß and mechanical load for 5 days. Subsequent tissue analysis for quantitative gene expression of metalloproteinases demonstrated a significant approximately 100-fold increase in MMP-13 expression as compared to the control (p = 0.003) (Fig 2). Upon administration of 1 mg/ml HA, the expression of MMP-13 was significantly decreased by about 67% (p = 0.01). The administration of 3 mg/ml HA resulted in a significantly reduced expression of approximately 37% as compared to the untreated sample (p = 0.015). There was no significant difference between the two HA concentrations.

**Fig 3:**
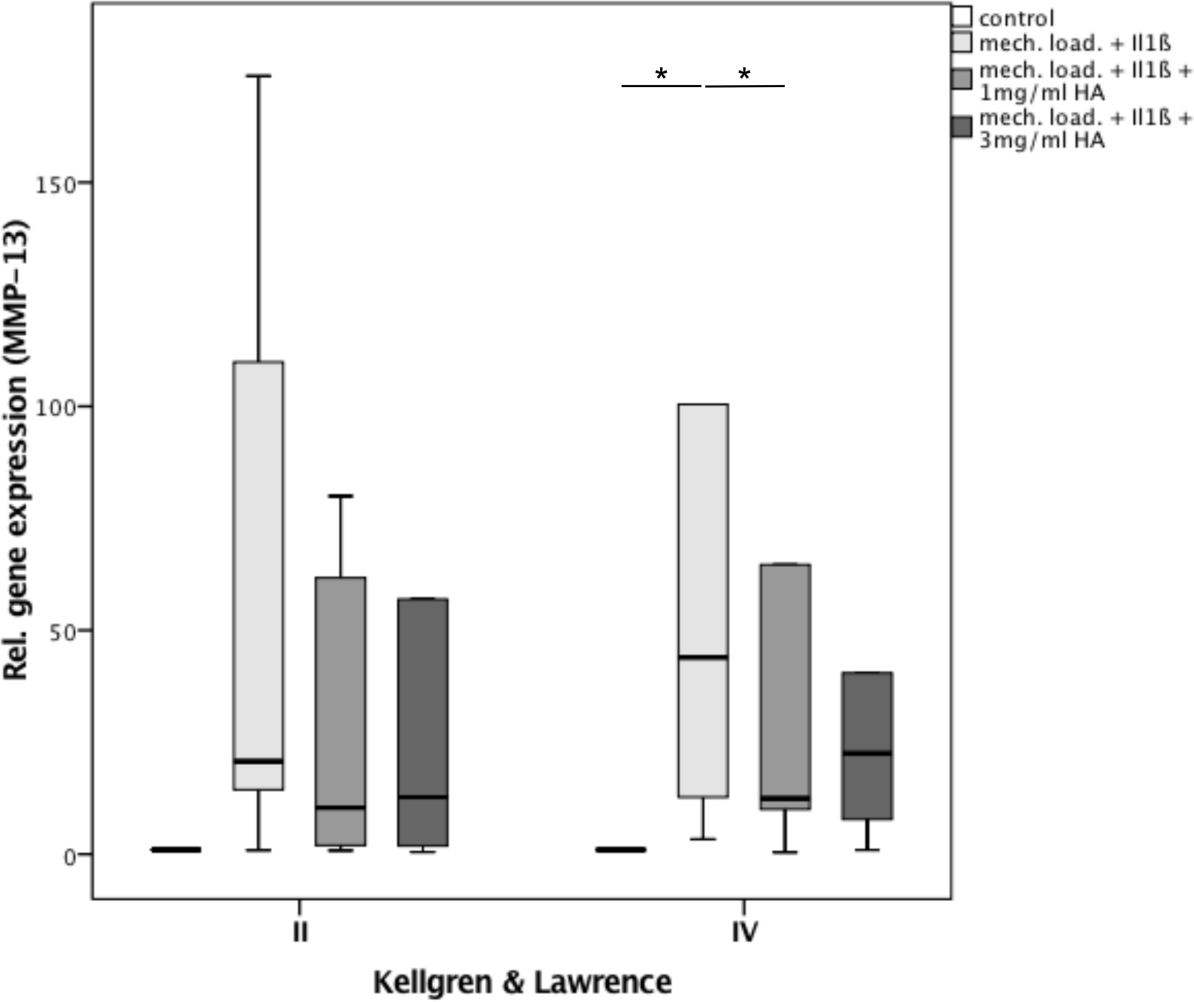
Separate analysis of relative gene expression of MMP-13 in all 3 study groups according to OA severity (K&L2 and K&L4): (1) 2ng/ml IL1ß + mechanical loading, (2) 2ng/ml IL1ß + mechanical loading + 1mg/ml HA, (3) 2ng/ml IL1ß + mechanical loading + 3mg/ml HA and the control; * indicates statistical significance with p<0,05.

Additionally, separate analysis of MMP-13 gene expression based on the K&L grade was performed. In K&L2 samples we observed a significant 57% increase of gene expression after stimulation with Il-1ß and mechanical load compared to the control (p = 0.046) (Fig 3). Administration of HA led to a reduction of MMP-13 gene expression by about 53% in the lower dose (1 mg/ml) and about 32% in the higher dose HA group without statistical significance (p = 0.075; p = 0.075). There was no significant difference between the two HA concentrations.

Analysis of K&L4 samples demonstrated a significant approximately 150-fold increase in MMP-13 gene expression by stimulation with IL1ß as compared to the control (p = 0.028) (Fig 3). Upon administration of 1 mg/ml HA a significant decrease in gene expression of about 73% (p = 0.046) was observed. The administration of 3 mg/ml HA showed a reduction of MMP-13 expression by approximately 74% (p = 0.075). Similar to the analysis of the K&L2 group above, no significant difference between the two HA concentrations was found.

**Fig 4:**
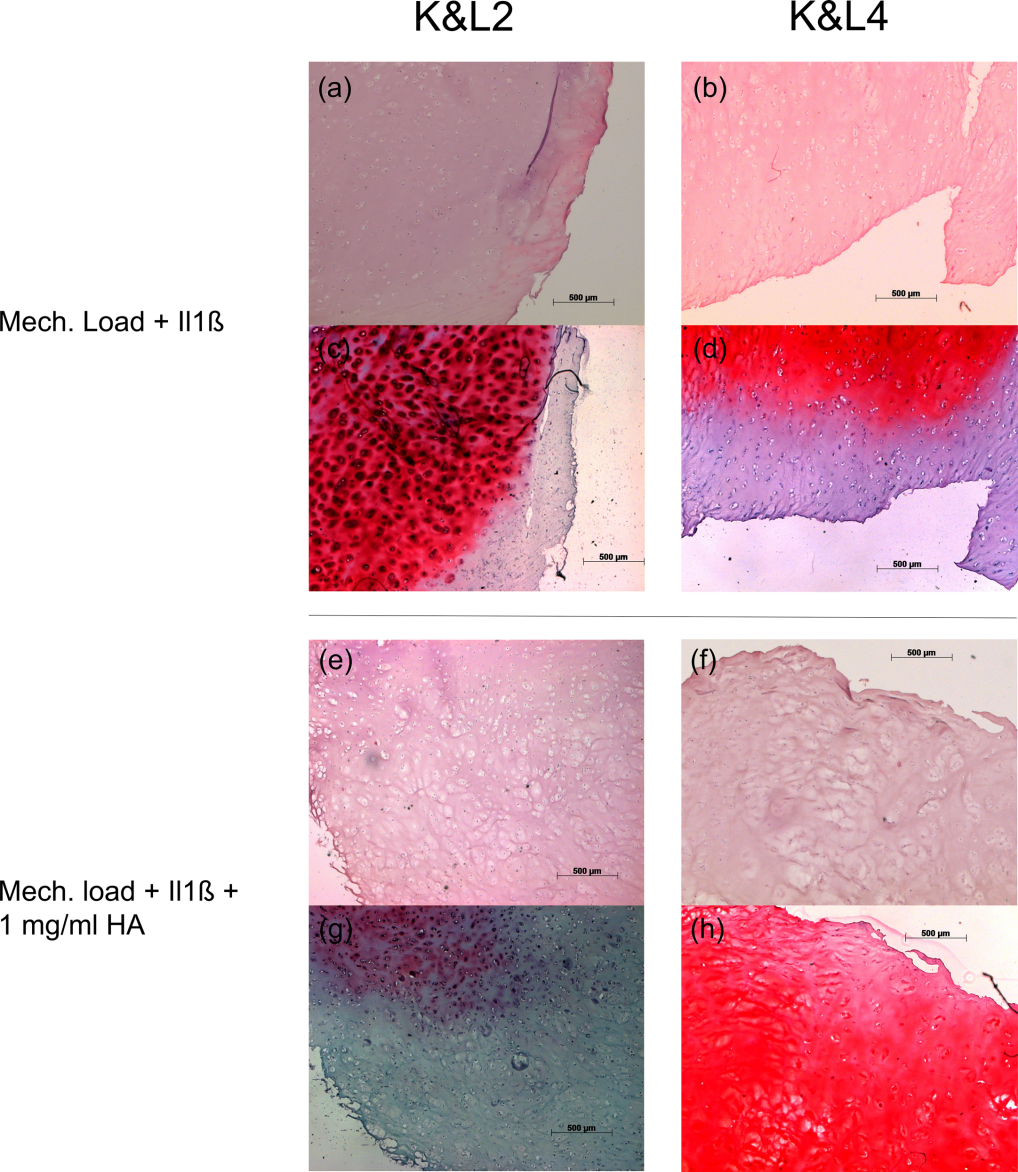
Photomicrographs of articular cartilage samples. (**a** and **b**) H&E stained sections of K&L2 and K&L4 cartilage samples upon stimulation with Il1ß and mechanical loading. (**c** and **d**) Safranin O stained sections of K&L2 and K&L4 cartilage samples upon stimulation with Il1ß and mechanical loading. (**e** and **f**) H&E stained sections of K&L2 and K&L4 cartilage samples upon additional administration of 1 mg/ml HA. (**g** and **h**) Safranin O sections of K&L2 and K&L4 cartilage samples upon additional administration of 1 mg/ml HA. Scale bar = 500 μm.

Because previous work in transgenic mice by Neuhold et al. has correlated MMP-13 expression to proteoglycan degradation, we performed histology to determine proteoglycan presentation (Fig 4) [29]. In K&L2 samples, we observed a decrease in proteoglycan content (Fig 4C and 4G), while in K&L4 specimens a strong increase in proteoglycan content was seen. (Fig 4D and 4H).

**Fig 5:**
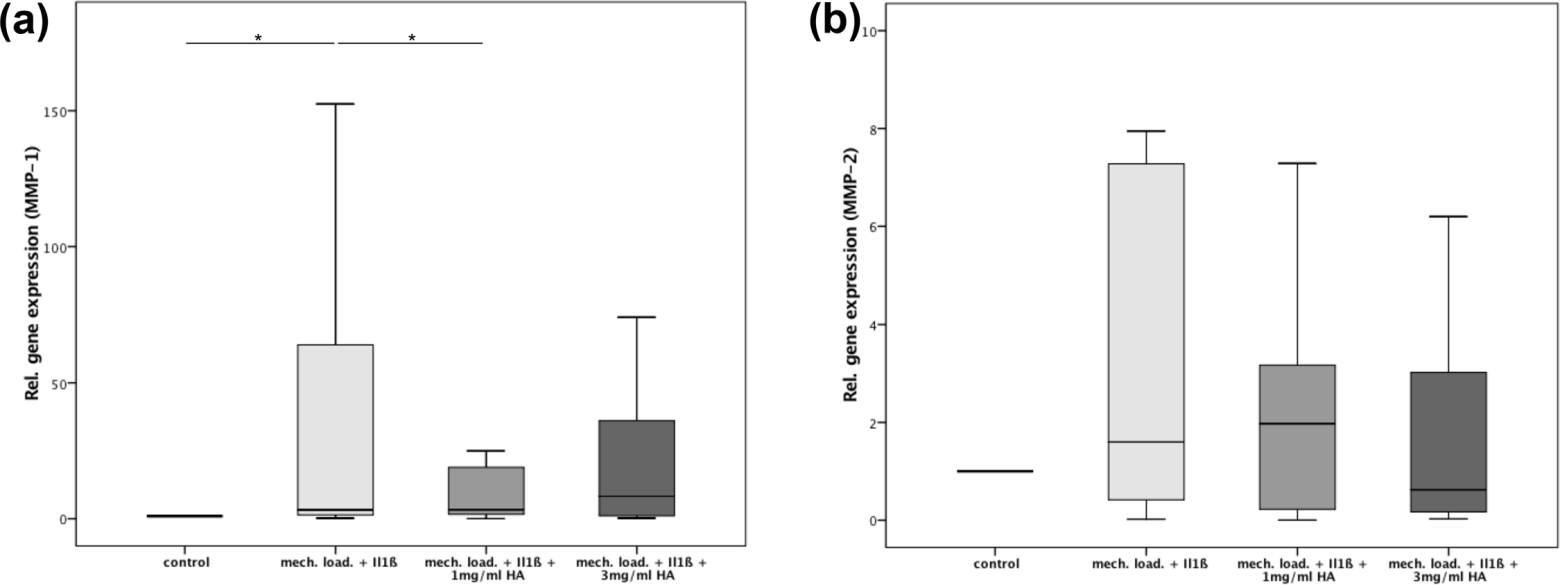
**(a)** Relative gene expression of MMP-1 in the 3 study groups: (1) 2ng/ml IL1ß + mechanical loading, (2) 2ng/ml IL1ß + mechanical loading + 1mg/ml HA, (3) 2ng/ml IL1ß + mechanical loading + 3mg/ml HA and the control; * indicates statistical significance with p<0,05; **(b)** Relative gene expression of MMP-2 in the 3 study groups: (1) 2ng/ml IL1ß + mechanical loading, (2) 2ng/ml IL1ß + mechanical loading + 1mg/ml HA, (3) 2ng/ml IL1ß mechanical loading + 3mg/ml HA and the control; * indicates statistical significance with p<0,05.

In addition to MMP-13 we also assessed gene expressions of MMP-1 and MMP-2. Analysis of MMP-1 showed a significant 36-fold increased gene expression after stimulation with IL1ß compared to the control group (p = 0.08) (Fig 5A). Subsequent administration of 1 mg/ml HA led to a significant decrease of MMP-1 gene expression by approximately 67% (p = 0.05). Upon application of HA in the higher concentration (3 mg/ml) no reduction of gene expression of MMP-1 could be observed (p = 0.43). Again, the difference between the HA groups was statistically not significant (p = 0.06).

Analysis of MMP-2 gene expression demonstrated an increase upon stimulation with IL1ß compared to the control group (p = 0.14) (Fig 5B). However, administration of 1 mg/ml or 3 mg/ml HA did not significantly influence gene expression of MMP-2.

## Discussion

In this study, the effect of HA on metalloproteinase expression was assessed in an in vitro model of OA tissue degradation exploring human cartilage specimens exposed simultaneously to a catabolic IL1β and anabolic mechanical stimulus. It was observed that under these conditions HA reduced the expression of MMP-13 and MMP-1.

To model the molecular events, such as MMP expression, of the OA joint in vitro is challenging. For example, the initial molecular alterations causing OA remain largely unknown. Further, during disease progression a variety of cartilaginous and bony tissues acquire pathological alterations. Moreover, disease progression is likely driven by a complex interplay of different catabolic and anabolic stimuli which exert negative and positive effects on the integrity of the joint tissue, respectively. Previous studies have already identified some of the important stimuli in OA. For instance, the cytokine Il1ß is an abundant catabolic driver in OA progression [30]. Increased levels of Il1ß are found in joint effusions of OA and also rheumatoid arthritis patients promoting an increase and decrease in ECM breakdown and formation, respectively [31]. This can induce a catabolic-anabolic imbalance leading to progressive breakdown of cartilage ECM [20, 31]. Among the anabolic stimuli in the OA joint is mechanical loading. In OA patients, moderate activity can have a positive impact on pain level and even more importantly progression of OA [32]. Consistently, studies mimicking the complex multi-axial motion patterns demonstrated a significant up regulation of the expression of ECM components in cartilage explants [23, 33, 34]. Furthermore, Jeon et al. observed an up regulated gene expression of aggrecan and collagen II in osteoarthritic chondrocytes after mechanical stimulation with dynamic compression for 2 weeks [35]. Therefore, for this study we decided to investigate MMP expression not in a model of sole catabolic stimulation but combined catabolic IL1β and anabolic mechanical stimulation. Compared to the unstimulated controls we found increased expression of all examined MMPs under these conditions. However, we have chosen both the Il1ß concentration and loading protocol based on previously reported data and have not further titrated them in our study. A titration of the stimuli should be considered in further work because it may permit a model in which the balance between catabolic and anabolic tissue response can be gradually adjusted and hence further investigated.

In the above model, addition of HA resulted in a significant reduction of MMP-13 and MMP-1 expression by over 60% upon administration of 1 mg/ml HA. These findings are comparable with the reported effect of HA on MMP expression under sole catabolic IL1β stimulation [19, 32]. Thus, our experiments confirm our hypothesis that under combined IL1β and mechanical stimulation HA exhibits a suppressive effect on the expression of metalloproteinases. Our analysis of MMP-13 expression based on the K&L grade, demonstrated a statistically significant effect of HA only in the severe OA samples with grade K&L4. Histological assessment of the proteoglycan content demonstrated a decrease and increase in K&L2 and K&L4, respectively. With respect to K&L4, this finding suggests that the HA-mediated inhibition of MMP-13 expression leads to a decrease of proteoglycan degradation. A finding consistent with the previous observation that forced overexpression of MMP-13 results in increased proteoglycan degradation [29]. Interestingly, clinical studies suggest a greater effect after HA administration in patients with moderate OA [5, 6].

The potential therapeutic value of HA suppression of MMP-13 expression has been demonstrated by Little et al. [36]. Studying surgically induced OA in a murine MMP-13 knockout model, they identified MMP-13 as one major contributor to OA progression. Importantly, they could show inhibited cartilage erosion in the MMP-13 deficient mice compared to wild type mice. With regard to higher concentrations of HA (3 mg/ml), we observed in our study less of a suppressive effect on MMP-1 expression. With Collagen II as the main substrate for MMP-1 it is notable that HA can also have potentially harmful effects in higher doses on osteoarthritic cartilage as suggested by Gonzales-Fuentes et al. [37]. In their study they observed increased urinary levels of Collagen type II C-telepeptide, a marker of Collagen II breakdown, after intraarticular injection of HA. Lastly, MMP-2 expression was not influenced significantly by the administration HA. These results are comparable to previously published studies indicating an only weak potential of external regulation of MMP-2 expression [38]. However, Jeon et al. could demonstrate a discrepancy between the MMP-2 expression and the actual amount of secreted, potentially limiting gene expression data with regard to MMP-2 [39].

## Supporting Information

S1 Data A minimal data set underlying our findings has been added as supplementary file. (XLSX)
